# Changes in Culture Expanded Human Amniotic Epithelial Cells: Implications for Potential Therapeutic Applications

**DOI:** 10.1371/journal.pone.0026136

**Published:** 2011-11-02

**Authors:** Gita Pratama, Vijesh Vaghjiani, Jing Yang Tee, Yu Han Liu, James Chan, Charmaine Tan, Padma Murthi, Caroline Gargett, Ursula Manuelpillai

**Affiliations:** 1 Centre for Reproduction & Development, Monash Institute of Medical Research, Monash University, Clayton, Victoria, Australia; 2 Centre for Inflammatory Diseases, Department of Medicine, Monash University, Clayton, Victoria, Australia; 3 Department of Obstetrics & Gynecology and The Ritchie Centre, Monash Institute of Medical Research, Monash University, Clayton, Victoria, Australia; 4 Department of Obstetrics & Gynecology, University of Melbourne and Department of Perinatal Medicine, Pregnancy Research Centre, Royal Women's Hospital, Melbourne, Victoria, Australia; University of Reading, United Kingdom

## Abstract

Human amniotic epithelial cells (hAEC) isolated from term placenta have stem cell-like properties, differentiate into tissue specific cells and reduce lung and liver inflammation and fibrosis following transplantation into disease models established in mice. These features together with their low immunogenicity and immunosuppressive properties make hAEC an attractive source of cells for potential therapeutic applications. However, generation of large cell numbers required for therapies through serial expansion in xenobiotic-free media may be a limiting factor. We investigated if hAEC could be expanded in xenobiotic-free media and if expansion altered their differentiation capacity, immunophenotype, immunosuppressive properties and production of immunomodulatory factors. Serial expansion in xenobiotic-free media was limited with cumulative cell numbers and population doubling times significantly lower than controls maintained in fetal calf serum. The epithelial morphology of primary hAEC changed into mesenchymal-stromal like cells by passage 4–5 (P4–P5) with down regulation of epithelial markers CK7, CD49f, EpCAM and E-cadherin and elevation of mesenchymal-stromal markers CD44, CD105, CD146 and vimentin. The P5 hAEC expanded in xenobiotic-free medium differentiated into osteocyte and alveolar epithelium-like cells, but not chondrocyte, hepatocyte, α- and β-pancreatic-like cells. Expression of HLA Class IA, Class II and co-stimulatory molecules CD80, CD86 and CD40 remained unaltered. The P5 hAEC suppressed mitogen stimulated T cell proliferation, but were less suppressive compared with primary hAEC at higher splenocyte ratios. Primary and P5 hAEC did not secrete the immunosuppressive factors IL-10 and HGF, whereas TGF-β1 and HLA-G were reduced and IL-6 elevated in P5 hAEC. These findings suggest that primary and expanded hAEC may be suitable for different cellular therapeutic applications.

## Introduction

Human amniotic epithelial cells (hAEC) line the inner of two fetal derived membranes attached to the placenta. hAEC arise from pluripotent epiblast cells of the embryo and are among the first cells to differentiate in the conceptus [1]. Studies have shown that even at term pregnancy, primary hAEC isolated from amnion membranes retain some of the features of their founder cells, expressing pluripotency associated genes and differentiating into lineages derived from each of the three primary embryonic germ layers *in vitro* [2], [3]. Primary hAEC also display similarities to multipotent mesenchymal stromal/stem cells (MSC) expressing some of the surface antigens defining MSC, and like MSC lack hematopoietic and monocytic lineage markers [4], [5], [6].

Importantly, primary hAEC have several features that make them most attractive for cellular therapies. Compared with adult tissue derived stem cells, hAEC are plentiful and obtained without invasive and expensive procedures from term placenta, a widely accepted non-controversial source of stem cells. Replacement of cells damaged by disease, injury and aging remains a key goal in many therapeutic applications. In this context, hAEC have been shown to differentiate into functional neurons in spinal cord injury models [7], [8], insulin secreting pancreatic β-islet like-cells that normalized blood glucose in diabetic mice [9] and recently into surfactant producing alveolar epithelial cells in the lung [6]. Therapies aimed at reducing tissue inflammation and scarring to promote host tissue repair are another important potential application of stem cells. Studies in murine models of lung and liver fibrosis have shown that primary hAEC reduce inflammation and fibrosis and induce tissue remodeling and repair [6], [10], [11]. Further, hAEC transplantation appears to be safe and tumour or teratoma formation has not been demonstrated in spite of Oct-4, Sox-2 and Nanog expression that are linked to teratoma formation by embryonic and induced pluripotent stem cells [2], [3], [6], [11].

Another key feature is that primary hAEC appear to be amenable to allogeneic transplantation and indeed have been successfully transplanted into non-related human recipients during trials for lysosomal storage diseases [12]. Successful transplantation across histocompatibility barriers is probably facilitated by low HLA Class IA antigen expression and absence of HLA Class II antigens [2], [4], [13], [14]. Primary hAEC have also been shown to exert potent immunosuppressive properties inhibiting T cell proliferation [13], [14], [15], although the mechanisms remain unclear.

Approximately 50–100 million hAEC can be harvested from each term amnion membrane [3], [16]. However, cellular therapies would require several billion cells from each cell line for multiple dosing regimens and, importantly, to prevent micro-chimerism and potential immune responses arising from cells that have been pooled from several unrelated donors. For clinical applications, large numbers of MSC are generated by serial expansion under xenobiotic-free conditions to comply with good manufacturing practices (GMP) [17]. hAEC do not appear to be amenable to extensive expansion in animal serum supplemented media. Expression of pluripotency genes was suppressed during expansion in fetal calf serum (FCS) accompanied by changes in phenotype and surface antigen expression suggestive of an epithelial to mesenchymal transition [4], [5]. While hAEC expanded in FCS differentiated into osteocytes *in vitro* [5], whether expanded cells retain the ability to differentiate into lineages having therapeutic potential, such as hepatocytes and pancreatic β-islet cells, remains unknown. Importantly, the immunogenicity, immunosuppressive and secretory properties of expanded hAEC are unknown. We investigated if hAEC could be expanded in xenobiotic-free media and compared the differentiation, immunophenotype and immunosuppressive properties of cells expanded in xenobiotic-free medium with FCS supplemented medium and primary cells. The findings showed that expansion led to significant differences in the expression of markers, capacity to differentiate, ability to suppress T cell proliferation and the secretion of immunosuppressive factors by hAEC.

## Materials and Methods

### Ethics Statement

The study was approved by Southern Health and Royal Women's Hospital Human Research Ethics Committees and Institutional Review Boards of Monash University and University of Melbourne. Informed, written consent was obtained from each patient prior to amnion tissue collection. Amnion membranes were retrieved from placentae delivered by healthy women with a normal singleton pregnancy undergoing elective cesarean section at term (37–40 weeks gestation) for breech presentation or prior section (n = 30). Membranes were collected in DMEM/F12 medium containing 100 U/ml penicillin, 100 µg/ml streptomycin, 0.25 µg/ml amphotericin B and 2 mM L-glutamine (Gibco, Grand Island, NY). Isolation of splenocytes from C57BL/6 mice was approved by the Animal Ethics Committee, Monash University (approval number MMCB 2009/16).

### Isolation and Characterization of hAEC

Cells were isolated using a method described previously [2]. Briefly, tissue was digested twice in 0.25% trypsin containing 0.5 mM EDTA in Hanks Balanced Salt Solution (HBSS) for 15 min at 37°C with gentle shaking. Trypsin was inactivated with newborn calf serum, solution filtered and centrifuged at 175×*g*. The cells were washed in DMEM/F12 and contaminating erythrocytes lysed in hypotonic solution (8% ammonium chloride, 0.84% sodium bicarbonate and 0.37% EDTA) for 10 min at 37°C. Media and reagents were purchased from Gibco. Purity of the isolates was determined by flow analysis for the epithelial marker cytokeratin-7 (CK7; Dako, Carpentaria, CA), as described earlier [2]. Isolates that were >99% positive for CK7 and exhibiting a cobblestone appearance in primary culture were used in the experiments described below. Each of the following studies were carried out on hAEC isolated from (n = 4–6) amnion membranes.

### hAEC Expansion

To determine if hAEC could be expanded under xenobiotic-free conditions, commercially available serum free media and human serum were tested. Freshly isolated hAEC (1.5×10^6^) were plated in 25 cm^2^ flasks and cultured in the following: 1) Epilife medium with xenobiotic-free S7 supplement (Cascade Biologics, Portland, OR); 2) PC-1 medium (Lonza, Walkersville, MD); 3) Stempro MSC medium (Gibco); 4) CnT22 medium (Millipore, Billerica, MA); and 5) 2–10% heat inactivated human serum (Gibco) in DMEM/F12. Comparisons were made against hAEC grown in DMEM/F12 with 10% FCS. Preliminary studies demonstrated better growth with addition of recombinant human epidermal growth factor (rhEGF). Therefore, all culture media were supplemented with 10 ng/ml rhEGF (Invitrogen, Carlsbad, CA). Primary cultures were designated as passage 0 (P0). Media were changed thrice weekly and cells passaged at ∼80% confluence using a split ratio of 1∶2. Cells were lifted using TrypLE (Gibco) and counted (Countess™ Automated Cell Counter, Invitrogen). Cumulative cell numbers (CCN) [4] and cumulative population doublings (CPD) were determined. CPD was calculated using the formula: CPD = [ln (cumulative cell number)]/ln 2 [18]. Where possible, cells were maintained until P7.

To monitor changes in morphology with expansion, P2 hAEC with cobblestone epithelial appearance were labeled with CFSE (Invitrogen) following manufacturer's instructions and then expanded. Cultures were passaged twice after reaching ∼80% confluence and photographed under an inverted fluorescence microscope (Olympus IX71, Melville, NY).

### Flow Cytometry

hAEC (approximately 1×10^5^ cells) suspended in 100 µl of PBS/2% FCS/0.01% sodium azide were incubated with directly conjugated or unconjugated anti-human primary antibodies or matched-isotype control IgG (Table 1) for 45 min at 4°C. After several washes cells were incubated with phycoerythrin (PE)-conjugated goat anti-mouse Ig F(ab′)_2_ fragments (10 µl/ml; Chemicon, Melbourne, Australia), Alexa Fluor (AF) 647-conjugated goat anti-mouse IgG (10 µg/ml; Molecular Probes, Eugene, OR) or AF 488-conjugated chicken anti-rat IgG (10 µg/ml) for 30 min at 4°C except for CD31, CD45 and CD90. Blocking serum (5 µl chicken serum for CD29 and CD49f; 5 µl goat serum for the balance) was also included during incubation with primary and secondary antibodies. Cells were analyzed by flow cytometry using Cyclops SUMMIT software (Version 5.0; Dako Cytomation, Fort Collins, CO).

**Table 1:** Antibodies Used to Phenotype hAEC by Flow Cytometry.

Primary Antibodies/Fluorochrome	Isotype	Working Concentration	Source of primary antibodies
E-cadherin	Mouse IgG1	(supernatant)	Hans-Jorg Buhring, Tϋbingen, Germany
CD49f	Rat IgG2a	5 µg/ml	BD Biosciences, San Jose, CA
EpCAM	Mouse IgG1	11.8 µg/ml	Dako, Glostrup, Denmark
CD44	Mouse IgG2b	1 µg/ml	BD Biosciences, San Jose, CA
CD90/APC	Mouse IgG1	1 µg/ml	BD Biosciences, San Jose, CA
CD105	Mouse IgG1	10 µg/ml	BD Biosciences, San Jose, CA
CD146	Mouse IgG2a	(supernatant)	Stem Cell Centre, Melbourne, Australia
Vimentin	Mouse IgG1	0.28 µg/ml	Invitrogen, Camarillo, CA
PDGFR-β	Mouse IgG1	20 µg/ml	R&D Systems, Minneapolis, MN
CD29	Rat IgG2a	1 µg/ml	BD Biosciences, San Jose, CA
CD45/FITC	Mouse IgG1	10 µg/ml	Caltag, Burlingame, CA
CD31/PE	Mouse IgG1	4 µg/ml	Dako, Glostrup, Denmark
HLA-A-B-C	Mouse IgG1	0.5 µg/ml	BD Biosciences, San Jose, CA
HLA-DR-DP-DQ	Mouse IgG2a	2 µg/ml	BD Biosciences, San Jose, CA
CD40	Mouse IgG2a	0.4 µg/ml	Abcam, Cambridge, UK
CD80	Mouse IgG1	4 µg/ml	Abcam, Cambridge, UK
CD86	Mouse IgG1	0.4 µg/ml	Abcam, Cambridge, UK

### Immunocytochemistry

hAEC cultured in 8-well chamber slides (2×10^4^ cells/well) were fixed in 4% paraformaldehyde for 20 min. Endogenous peroxidase activity was quenched in 0.3% H_2_O_2_ (Orion Laboratories, Balcatta, Australia) in methanol and non-specific binding blocked in PBA solution (Shandon, Pittsburgh, PA) for 15 min. Cells were incubated with antibodies against HLA-G (1∶100; BD Biosciences, San Jose, CA) or CK7 (1∶100; Dako) in DPBS containing 0.2% Triton X-100 for 1 h at 37°C. Negative controls were incubated with mouse IgG_1_,_k_ (BD Biosciences). Cells were washed and incubated with anti-mouse-biotinylated secondary antibody (1∶200; Vector Laboratories, Burlingame, CA) for 30 min followed by ABC kit reagents (Vector Laboratories). Immunostaining was visualized using DAB chromogen (Sigma-Aldrich, St. Louis, MO).

### Karyotype Analysis

Chromosomal analysis using G-banding was performed by Southern Cross Pathology, Monash Medical Centre. After 4–6 h treatment in colchicine, cells were harvested and fixed in methanol/acetic acid. G-Bands were visualized by 0.025% trypsin (BD Difco, Sparks, MD) treatment for 5–20 sec, followed by incubation in 0.04% Leishman's stain (Sigma-Aldrich, Castle Hill, Australia) for 5 min.

### Clonal Culture

P0 hAEC have been shown to form clonal colonies [2]. To determine if P5 hAEC were clonogenic, cells were seeded at low density (∼30–50 cells/cm^2^ in 100 mm diameter petri dishes). Cultures were maintained in DMEM/F12 with 10% FCS or Epilife with 10 ng/ml rhEGF for up to 21 d with media replaced once weekly. Clusters containing more than five cells were considered to be colonies. Cloning efficiency was calculated using the formula: cloning efficiency (%) = (number of colonies/number of cells seeded)×100 [2].

### Alkaline Phosphatase Activity

hAEC were seeded in 8-well chamber slides (2×10^4^/well) and fixed in 4% paraformaldehyde for 1–2 min at room temperature (RT). Alkaline phosphatase activity was detected using a kit (Millipore), following manufacturer's instruction and then counterstained with haematoxylin for 10–15 sec. A human embryonal carcinoma (hEC) cell line (provided by Prof. Martin Pera, University of Southern California, USA), served as a positive control [19].

### Transmission Electron Microscopy (TEM)

Cultures were fixed in 2.5% glutaraldehyde for 2 h at RT, washed in 0.1 M cacodylate buffer and post-fixed in 1% osmium tetroxide for 2 h. Cells were dehydrated through ethanol, infiltrated, embedded in resin-araldite mixture and polymerized at 60°C for 24 h. Ultrathin sections (90 nm) were stained with uranyl acetate for 10 min and Reynolds lead for 2 min. Sections were viewed on Hitachi H-7500 (Tokyo, Japan) transmission electron microscope and images acquired digitally.

### Differentiation and Characterization

P0 and P5 hAEC were plated in 8-well chamber slides (2×10^4^ cells/well) or 6-well plates (2.5×10^5^ cells/well). Cells were cultured in Small Airway Growth Medium (SAGM; Lonza, Walkersville, MD; Table 2) to induce differentiation into alveolar epithelium-like cells [6]. Supplements listed in Table 2 were added to basal medium to differentiate cells into mesodermal and endodermal derived lineages. Chondrocytic differentiation was induced by pelleting hAEC (3×10^5^ cells) and adding supplements (Table 2). Treated cultures and non-stimulated controls in basal medium were maintained for up to four weeks with media changes thrice weekly.

**Table 2:** Differentiation media and supplements.

Lineage	Differentiation medium/supplements/duration/references	Characterized by
Endodermal		
Alveolar Epithelium	SAGM medium containing hydrocortisone, BSA-fatty acid free serum, bovine pituitary extract, rhEGF, epinephrine, transferrin, insulin, retinoic acid and tri-iodothyronine. Four weeks [6].	Prosurfactant Protein-C
Pancreatic	Nicotinamide (10 mM), retinoic acid (1 mM), N2 supplement, rhEGF (10 ng/ml), exendin-4 (10 nM). Two weeks [42].	Insulin/glucagon (GCG)
Hepatic	rhEGF (10 ng/ml, 5 days), then dexamethasone (0.1 µM), insulin (0.1 µM) for 3 weeks [2], [3].	Hepatocyte Nuclear Factor-4α, Albumin
Mesodermal		
Osteocytic	1,25-Dihydroxyvitamin D3 (0.01 µM), ascorbic acid (50 µM), b-glycerophosphate (10 mM). Four weeks [2].	Alizarin Red Stain
Chondrocytic	Insulin (6.25 µg/ml), ascorbic acid-2-phosphate (50 µM), transforming growth factor-β1 (10 ng/ml). Four weeks [43].	Alcian Blue

Secreted insulin was measured by Southern Cross Pathology, Monash Medical Centre using the Access/DXI Ultrasensitive Insulin assay (Beckmann-Coulter, Sydney, Australia). Immunocytochemistry was carried out to identify the hormone glucagon (GCG), produced by pancreatic α-cells. hAEC were fixed with 4% paraformaldehyde, endogenous peroxidase activity quenched in methanol and non-specific binding blocked in PBA. Cells were incubated anti-GCG (1∶50; R&D Systems; Minneapolis, MN) in PBS containing 0.2% Triton-X, overnight at 4°C. Mouse IgG2a (Dako) was applied to negative controls. Antibody binding was detected with DAB.

Differentiation into alveolar epithelium-like cells was also tested by immunocytochemistry for prosurfactant protein-C (proSP-C; Millipore). hAEC were fixed in ethanol and incubated with antibody against proSP-C (1∶200) in PBS containing 0.01% Tween 20, overnight at 4°C. Rabbit IgG (Dako) was applied to negative controls.

Differentiation into hepatocyte-like cells was tested by immunofluorescence for hepatocyte nuclear factor-4α (HNF-4α; Cell Signaling Technology, Danvers, MA) and albumin (R&D Systems). hAEC were fixed in ethanol and incubated with antibodies against HNF-4α (1∶6000) in PBS containing 0.01% Tween 20, overnight at 4°C. Controls were incubated with corresponding concentration of rabbit IgG. Antibody binding was detected using AF 488-conjugated goat anti-rabbit secondary antibody (1∶1000; Molecular Probes). Albumin antibody (1∶10 in 0.1% triton X-100) was applied overnight at 4°C. Mouse IgG2a was added to controls. Binding was detected using goat anti-mouse AF-568 (1∶1000; Molecular Probes). Cells were washed and mounted in Vectorshield containing DAPI nuclear stain (Vector Laboratories).

Osteocytic differentiation was assessed by identifying calcium deposition using Alizarin red staining. Cells were fixed in 10% neutral buffered formalin for 15 min at RT, washed twice in distilled water and incubated in 1 ml of 1% of Alizarin red (pH 4.1). Cultures were washed with distilled water and dried. Chondrocytic differentiation was assessed by Alcian blue staining. Cell pellets were fixed in 10% formalin for 1 h and embedded in paraffin. Sections were incubated in 1% Alcian blue in 0.1 M hydrochloric acid for 30 min, dehydrated and mounted in DPX.

### T cell Proliferation Assay

To compare the immunosuppressive properties of P0 and P5 hAEC, T cell proliferation assays were carried out as described previously [20]. In brief, splenocytes from C57BL/6 mice were seeded in 96-well plates (5.0×10^5^ cells/well) in complete RPMI 1640 medium (Gibco) supplemented with 10% FCS. P0 and P5 hAEC were irradiated (20 Gy) and added to splenocytes in different hAEC stimulator : splenocyte responder cell ratios. Then, 10 µg/ml Concanavelin A (Con A) was added to each well. Con A stimulated splenocytes minus hAEC served as a positive control. After 72 h incubation at 37°C in 10% CO_2_, 2 µCi [^3^H]-thymidine in a volume of 20 µl was added to each well and incubated for a further 24 h. Cells were harvested using a Packard Micromate 196-cell harvester (Packard Biosciences, Meriden, CT) and incorporated [^3^H]-thymidine measured using a Packard Tri-Carb 1900TR liquid scintillation analyzer. Measurements were taken from triplicate wells from each sample tested. Data were expressed as the percentage suppression of [^3^H]-thymidine uptake relative to Con A stimulated splenocytes.

### Measurement of Cytokines and Growth Factors

The production of cytokines and growth factors associated with immunosuppression [interleukin-(IL)-6, IL-10, transforming growth factor (TGF)-β and hepatocyte growth factor (HGF)] by P0 and P5 hAEC were measured using ELISAs (R&D Systems) following manufacturer's instructions. Samples were assayed in duplicate. The co-efficients of variation between sample duplicates was <8%.

### Migration Assay

To compare the migratory properties of P0 and P5 cells, hAEC were seeded in 6-well plates (2.5×10^5^ cells/well) and maintained in DMEM/F12+10% FCS or Epilife until confluent. Cross shaped scratch wounds were made using plastic pasteur pipettes and cultures washed several times to remove the dislodged cells. Cell migration over the wound areas was observed using phase contrast microscopy and images captured at regular intervals.

To investigate if CXCR4 played a role in migration, P0 and P5 hAEC were plated in 8-well chamber slides (2.0×10^4^ cells/well), fixed in methanol for 5 min and incubated with Image-iTTM FX signal enhancer (Molecular Probes) for 30 min at RT. Primary antibody against CXCR4 (1∶200; Abcam, Cambridge, UK) was applied and left for 1 h at RT. Corresponding concentration of goat serum was added to the negative controls. AF 488-conjugated rabbit anti-goat secondary antibody (1∶1000) was applied for 1 h at RT. Cells were washed and mounted in Vectorshield containing DAPI nuclear stain.

### Statistical Analysis

Data are shown as mean±SEM and analyzed using ANOVA followed by Tukey's post hoc and paired comparisons by the Student's t test (GraphPad Prism software, v5.02, San Diego, CA). Significance was accorded when p<0.05.

## Results

### hAEC Expansion in Xenobiotic Free Media

**Figure 1:**
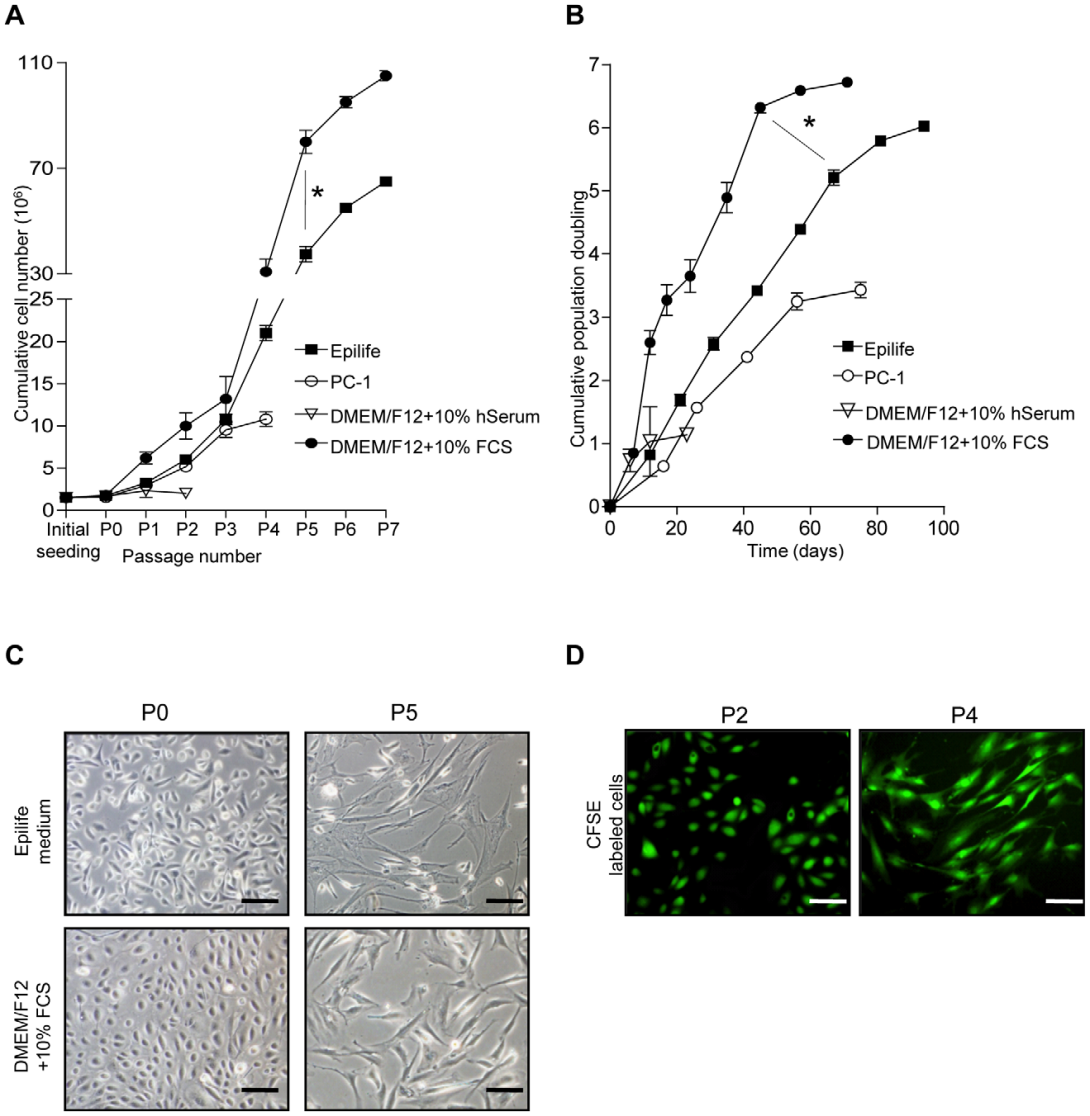
Expansion of hAEC. The hAEC grew well in the xenobiotic-free media Epilife until passage 5 (P5) and plateaued thereafter. A similar trend was seen in control cultures grown in DMEM/F12+10% FCS but the cumulative cell number in Epilife was lower than controls (*p = 0.002; **A**). Cumulative population doubling of hAEC cultured in Epilife media was lower than DMEM/F12+10% FCS (*p = 0.0026; **B**). hAEC appeared stromal-like at P5 in Epilife and DMEM/F12+10% FCS (**C**). Stromal-like cells at P4 retained the CFSE label suggesting the stromal cells arose from the labeled P2 epithelial cells (**D**). Scale bars = 100 µm.

To investigate whether hAEC could be expanded under xenobiotic supplement free conditions, the cells were cultured in commercially available serum free media and human serum supplemented with rhEGF. hAEC were readily expanded until P5 and plateaued thereafter in Epilife culture medium supplemented with S7 additive (Fig. 1A). A similar trend was seen in control cultures expanded in DMEM/F12 with 10% FCS and rhEGF. Cultures expanded in PC-1 media showed signs of senescence by P4. The cells failed to grow in serum free DMEM/F12, CnT22 and Stempro MSC media. hAEC maintained in DMEM/F12 containing 2–10% human serum could not be expanded beyond P2.

After an initial seeding density of 1.5×10^6^ cells, the CCN in Epilife reached 37.44±2.95×10^6^ cells by P5 but was significantly lower than cultures grown in DMEM/F12 containing 10% FCS (80±4.33×10^6^ cells; p = 0.002; Fig. 1A). In PC-1 and 10% human serum the CCN reached 10.80±0.88×10^6^ and 2.04±0.12×10^6^ cells by P4 and P2, respectively. The time needed for hAEC to reach P5 in Epilife was 64.75±1.6 d compared with 45.50±2.02 d in DMEM/F12 containing 10% FCS (p<0.0001). The CPD of 5.21±0.12 for hAEC cultured in Epilife by P5 was also significantly lower than in DMEM/F12 with 10% FCS (6.32±0.08; p = 0.0026; Fig. 1B).

During expansion, the cells changed their phenotype from a typical epithelial morphology in P0–P2 to transitional epithelial-stromal cells in P3–P4 and completely stromal-like cells by P5 in Epilife and DMEM/F12 containing 10% FCS (Fig. 1C). Cells cultured in PC-1 were stromal like at P1 (data not shown). We labeled P2 hAEC growing in Epilife and DMEM/F12+10% FCS showing typical epithelial cobblestone morphology with the intracellular dye CFSE to investigate whether the stromal-like cells at P4 would retain the dye. At P4, the stromal-like cells were labeled with CFSE (Fig. 1D), suggesting that these cells arose from the epithelial cells.

**Figure 2:**
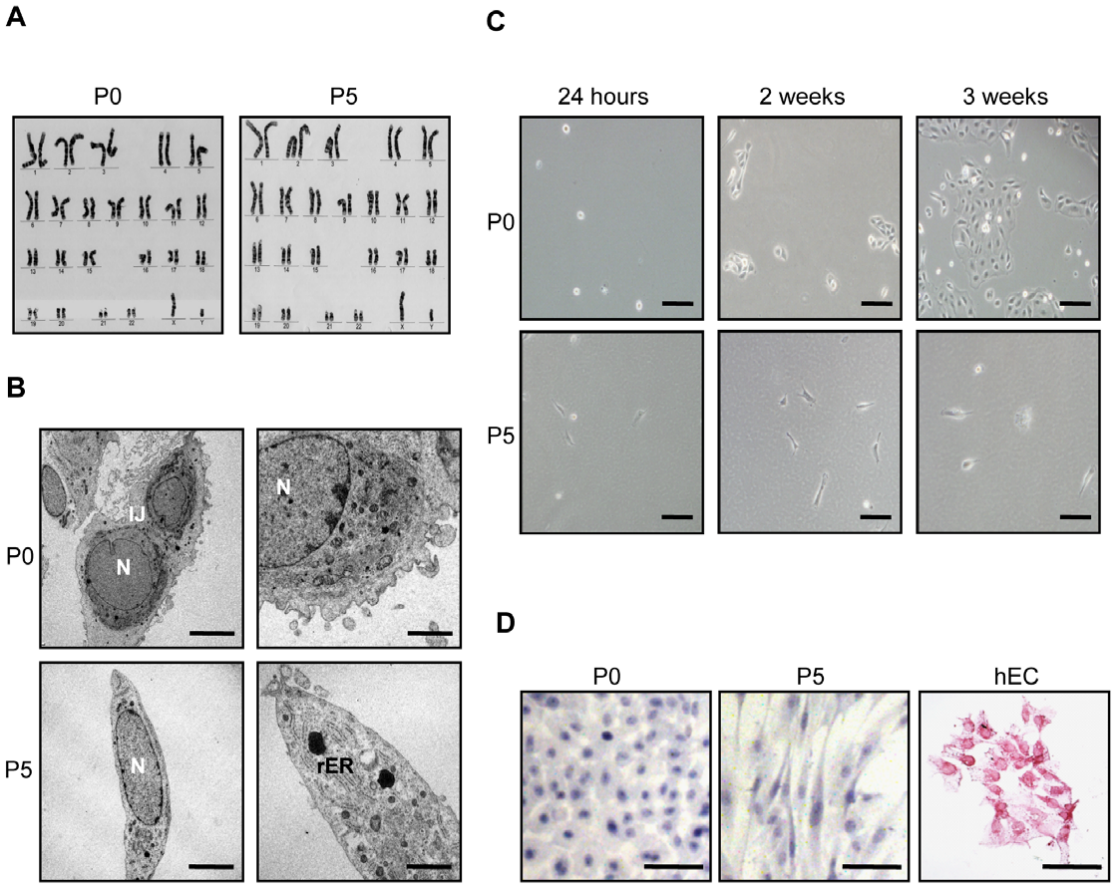
Features of cultured primary (P0) and cells expanded in Epilife medium to passage 5 (P5). Normal karyotype of P0 hAEC was retained at P5 (**A**). Transmission electron micrograph of P0 hAEC showed intercellular junctions (IJ), a multiloculated peripheral appearance compared to P5 hAEC. P5 cells had extensive rough endoplasmic reticulum (rER) and fewer cell surface projections. P0 and P5 cells showed high nuclear (N) to cytoplasmic ratio (**B**). P0 hAEC seeded at low density formed clonal colonies unlike P5 hAEC (**C**). hAEC lacked alkaline phosphatase activity unlike human embryonal carcinoma (hEC) cell line used as a positive control (**D**). Scale bars = 100 µm (**A–B**); 5 µm and 1 µm (**C**).

We compared features of P5 cells expanded in Epilife and DMEM/F12+10% FCS (DF) with the P0 cells. Analyses of P0 cells cultured in Epilife and DF showed no significant differences for any of the parameters tested; hence data for cultures grown in DF are shown in Figs. 2–6.

**Figure 3 pone-0026136-g003:**
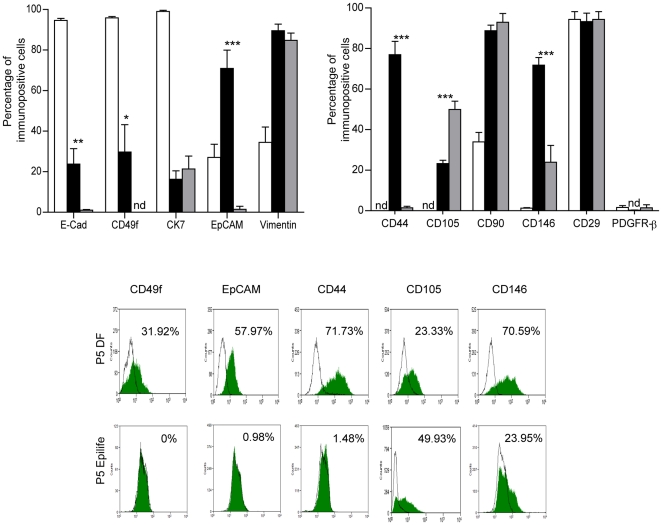
Phenotype of primary (P0) and passage 5 (P5) hAEC. Cells expressing epithelial markers E-cadherin, CD49f and CK7 declined with expansion while percentage of cells with MSC associated markers CD90, CD146 and the stromal marker vimentin were elevated in P5 cells grown in Epilife and DMEM/F12+10% FCS (DF). However, notable differences were also found between Epilife and DF expanded cells. Representative flow cytometry plots of markers that differed are shown. Open bars = P0, black shaded bars = P5 DF and grey bars = P5 Epilife. *p<0.05; **p<0.01; ***p<0.0001 by ANOVA and Tukey's post hoc test.

**Figure 4 pone-0026136-g004:**
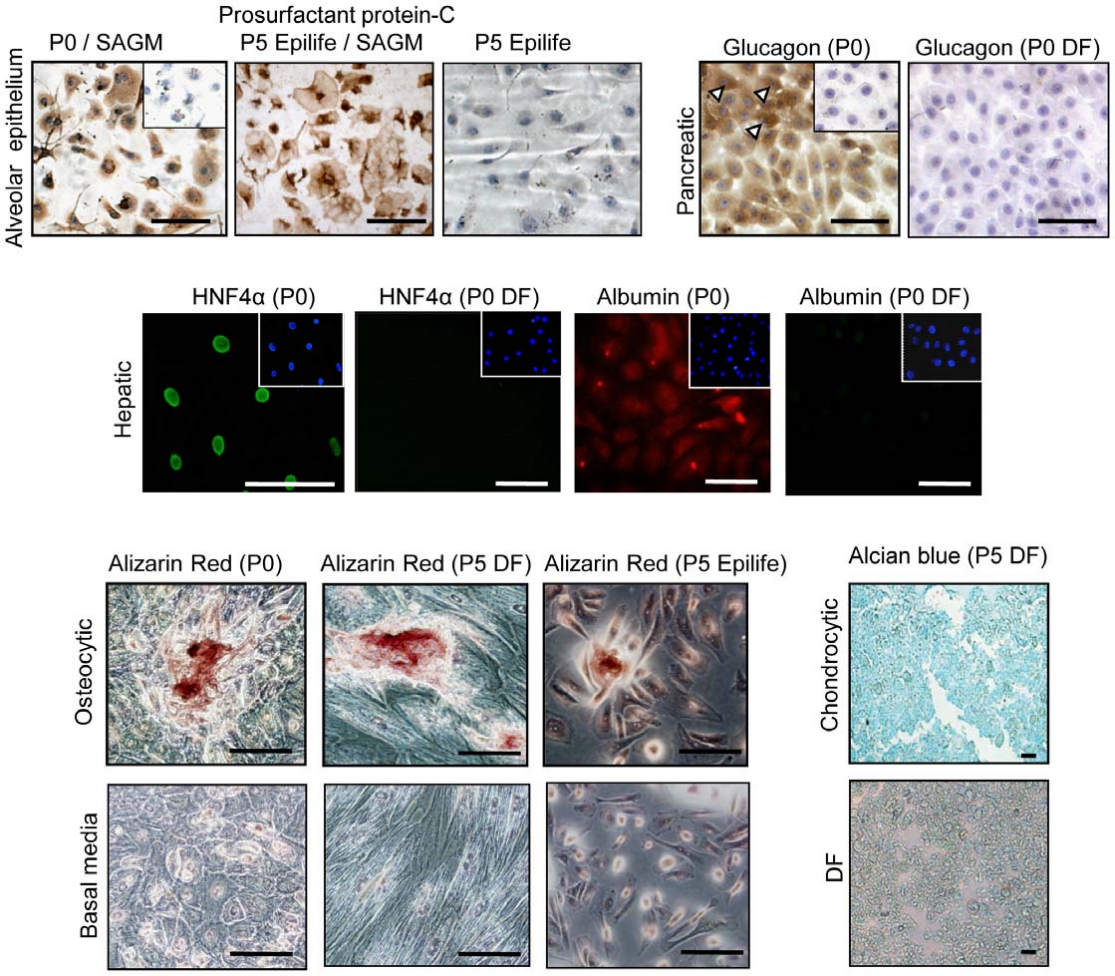
Differentiation of hAEC and their characterization. Primary (P0) and Epilife expanded passage 5 (P5) hAEC grown in SAGM produced prosurfactant protein-C characteristic of type 2 alveolar epithelial cells. Control P5 cultures maintained in Epilife lacked staining. Stimulated P0 hAEC contained glucagon (GCG), found in alpha pancreatic cells (arrow heads). Glucagon was absent in control cultures grown in DMEM/F12 medium with 10% FCS (DF). Inserts within panels show isotype controls (upper panel). P0 hAEC induced with supplements expressed hepatocyte nuclear factor-4α (HNF-4α) and albumin, unlike control cultures grown in DF. Cell nuclei stained with DAPI are shown in the inserts (middle panel). Alizarin red staining indicating calcium deposition characteristic of osteocytes in stimulated P0 and P5 cultures. Cartilage proteoglycans stained with Alcian blue in stimulated P5 DF expanded hAEC. Non-stimulated control cultures maintained in basal medium lacked evidence of differentiation into osteocyte and chondrocyte-like cells (lower panel). Scale bars = 100 µm.

**Figure 5 pone-0026136-g005:**
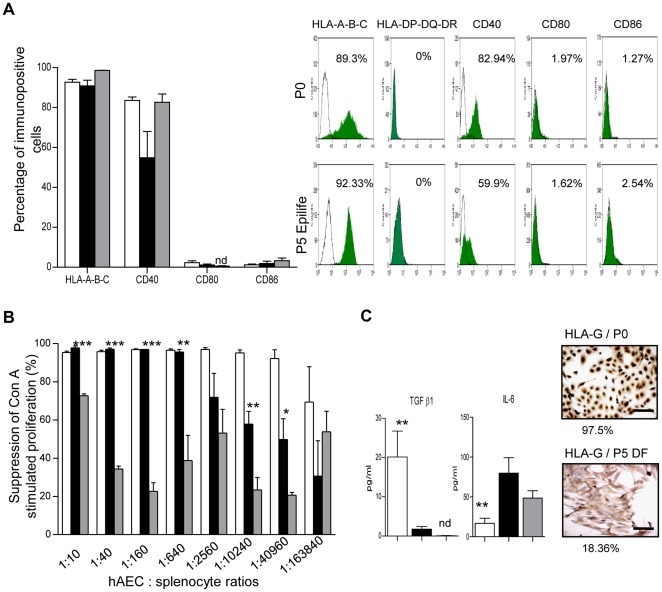
Immunogenicity of hAEC and effects on T cell proliferation. Primary (P0) and passage 5 (P5) hAEC expressed HLA-A-B-C but lacked HLA-DP-DQ-DR. Co-stimulatory molecules CD40, CD80 and CD86 remained unaltered at P5. Representative flow plots for HLA antigens and co-stimulatory molecules are shown (**A**). P0 and P5 hAEC suppressed Concanavelin A stimulated splenocytes from C57/BL6 mice, however P5 cells showed reduced suppression at higher splenocyte ratios (**B**). P0 and expanded cells produced the immunosuppressive factors TGF-β and IL-6. HLA-G that was abundant in P0 cells declined significantly in P5 grown in DMEM/F12+10%FCS (DF), while hAEC expanded in Epilife lacked HLA-G. Open bars = P0, black shaded bars = P5 cells expanded in DF and grey bars = P5 cells expanded in Epilife. Scale bars = 100 µm. *p<0.05; **p<0.01 and ***p<0.001.

**Figure 6:**
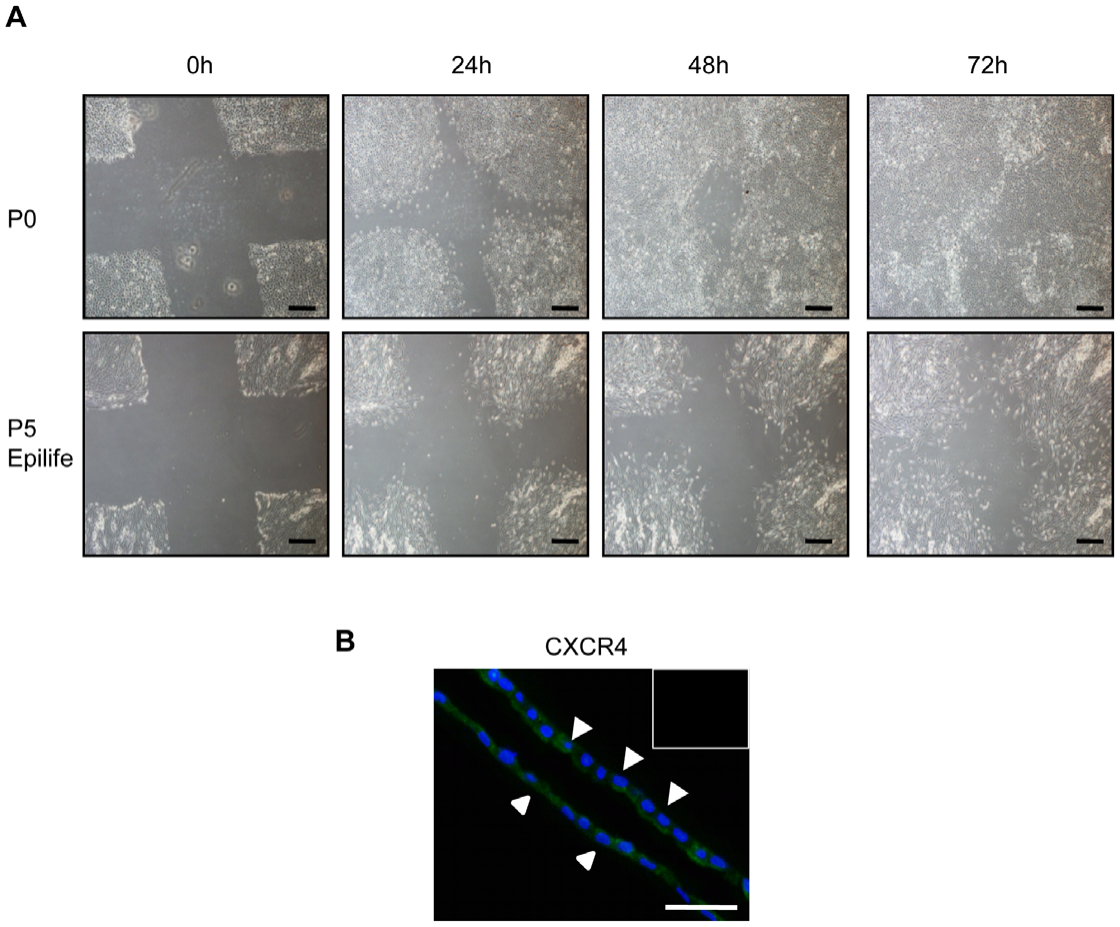
Cell migration assay. Primary, passage 0 (P0) hAEC migrated more rapidly compared with expanded passage 5 (P5) cells in a scratch wound assay (**A**). Cultured P0 and P5 cells lacked the chemokine receptor CXCR4 that has been implicated in cell migration (data not shown), however hAEC lining term delivered amnion membranes were immunopositive for CXCR4 (white arrows head = hAEC; **B**). Insert within panel shows staining of isotype control. Scale bars = 500 µm (**A**) and 100 µm (**B**).

### Properties of Expanded hAEC

Karyotype analyses were carried out to determine if chromosomal abnormalities arose during expansion. P5 cells expanded in Epilife and DF were found to retain the normal autosomal and XX or XY sex chromosome complement of the P0 cells (Fig. 2A).

We also compared the ultrastructural features of the P0 and expanded cells by TEM. P0 and P5 hAEC had a high nuclear : cytoplasmic ratio and prominent nucleoli (Fig. 2B). P0 hAEC were typically roundish cells with surface microvilli and cytoplasmic blebs. A multiloculated peripheral appearance with well developed intercellular junctions, particularly desmosomes and small amounts of rough endoplasmic reticulum (rER) were also seen in the P0 cells. In contrast, P5 hAEC expanded in Epilife and DF had extensive rER and associated Golgi complexes and very few surface villi compared to P0 cells.

Investigating clonal colony formation, small clusters containing >5 cells were observed in P0 cultures seeded at low density within two weeks (Fig. 2C). Large clonal colonies were evident by three weeks. The percentage cloning efficiency of P0 cells was 1.89±0.17. Cells from the primary epithelial colonies were sub-cloned 3–4 times but changed into stromal-like cells (data not shown). The expanded P5 hAEC failed to form clonal colonies.

Next, we tested for alkaline phosphatase activity. The P0 and P5 hAEC lacked alkaline phosphatase activity measured using a commercial assay, unlike the hEC cell line used as a positive control (Fig. 2D).

### Expression of Epithelial and Mesenchymal Markers with hAEC Expansion

Given the phenotypic changes observed with expansion, we ascertained if there were changes in epithelial (E-cadherin, CD49f, CK7, EpCAM), stromal (vimentin) and MSC associated markers (CD44, CD90, CD105, CD146, PDGFR-β, CD29). P5 DF-hAEC had reduced numbers of cells with epithelial markers compared to P0 (E-cadherin, CK7 and CD49f; P<0.001; Fig. 3A), and more cells with stromal/MSC markers (vimentin, CD90, CD146, CD44 and CD105; P<0.001). P5 Epilife cells also expressed stromal markers but were notably different to P5 DF-hAEC with lower percentage of CD44 and CD146 (P<0.0001) and higher numbers of CD105 positive cells (p<0.0001). Epithelial markers E-cadherin, CD49f and EpCAM were also reduced in P5 Epilife hAEC (P<0.01 compared to P5 DF-hAEC).

The percentage of CD29 positive cells was unaltered with expansion while PDGFR-β was low in P0 and P5 cells (Fig. 3A). Further, endothelial (CD31) and hematopoietic (CD45) markers were absent in P0 and expanded cells.

### Differentiation of hAEC and Characterization

We compared the differentiation potential of P0 hAEC and cells expanded in Epilife and DF into endodermal (alveolar epithelial cells, pancreatic cells, hepatocytes) and mesodermal (osteocytes, chondrocytes) lineages. P0 and P5 Epilife hAEC grown in SAGM medium (Table 2) produced proSP-C, a protein specific to type 2 alveolar epithelial cells (Fig. 4). However, the DF-P5 cells failed to grow in SAGM medium. P0 hAEC induced to differentiate into pancreatic-like cells secreted insulin (75.8±38.mIU/L). Some of the cells also stained positively for the hormone glucagon (GCG, Fig. 4). Neither insulin secretion nor GCG staining were detected in P5 cultures. Nuclear staining of the hepatocyte specific transcription factor HNF-4α and albumin was used to assess the differentiation of hAEC into hepatocyte-like cells. P0 hAEC stimulated with EGF followed by insulin and dexamethasone showed nuclear HNF-4α and albumin staining (Table 2; Fig. 4). However, expanded cells did not show evidence of differentiation into hepatocyte-like cells. In contrast, P0 and P5 hAEC maintained in osteocytic differentiation medium showed Alizarin red stained calcium deposits suggesting differentiation into osteocyte-like cells (Fig. 4). P0 and P5 Epilife hAEC failed to aggregate and did not differentiate into chondrocyte-like cells, whereas DF-P5 cells produced cartilage proteoglycans that were detected by Alcian blue staining (Fig. 4). Staining was absent in control cultures that were maintained in basal media minus the supplements shown in Table 2.

### Immunophenotype and Immunosuppressive Properties

We investigated if expansion induced changes in the expression of HLA Class I and II antigens and the co-stimulatory molecules. In excess of 90% of P0 and P5 cells expressed low to moderate levels of HLA-A-B-C (Fig. 5A). HLA-DP-DQ-DR was absent in primary and expanded cells. The co-stimulatory molecule CD40 was expressed at low levels by the majority of P0 and P5 hAEC tested (Fig. 5A). Very low levels of CD80 and CD86 were detected in <10% of P0 and P5 hAEC (Fig. 5A).

We also compared the immunosuppressive properties of the primary and expanded cells. P0 and P5 hAEC suppressed the proliferation of splenocytes from C57BL/6 mice stimulated with Con A (Fig. 5B). However, the P0 cells were highly suppressive over a much wider range of hAEC stimulator : splenocyte responder cell ratios, unlike the P5 hAEC that had significantly reduced ability to inhibit T cell proliferation at higher splenocyte ratios (p<0.01; Fig. 5B).

### Production of Immunosuppressive Factors

Secretion of factors (TGF-β1, IL-6, IL-10 and HGF) that have been shown to suppress T cell proliferation was measured. Primary and expanded hAEC secreted TGF-β1 and IL-6, but not IL-10 and HGF. However, significant differences were seen between P0, DF-hAEC and Epilife expanded hAEC in TGF-β1 and IL-6 production (Fig. 5C). HLA-G, a non classical Class IB antigen with restricted expression is also known to exert anti-inflammatory properties by suppressing T and Natural Killer cell activity. While P0 hAEC were HLA-G positive, the number of HLA-G producing cells decreased significantly with expansion in DF and were notably absent in cells expanded in Epilife (p<0.0001). MCP-1 is known to regulate monocyte chemotaxis. P0 and Epilife expanded hAEC did not secrete MCP-1 whereas P5 DF hAEC secreted substantial amounts of MCP-1 (mean±sem = 866.5±80.42 pg/ml).

### Migratory Properties

Cell migration to inflamed and damaged tissue sites is also an important feature of MSC. We investigated the migratory capacities of the P0 and expanded cells using a standard scratch wound assay. P0 hAEC migrated into the wound within 24 h and completely obliterated the scratch wound by 72 h. The DF and Epilife expanded hAEC showed reduced migration with the scratch wound still visible after 72 h (Fig. 6A). The chemokine receptor CXCR4 has been widely implicated in regulating the migration of MSC in response to CXCL12 [21]. Immunolocalization studies showed that CXCR4 was absent in the cultured P0 and expanded P5 cells. However, hAEC lining amnion membrane stained positively for CXCR4 (Fig. 6B).

## Discussion

We showed that hAEC can be expanded in xenobiotic-free media, but that cell expansion was limited and that the hAEC underwent phenotypic changes consistent with an epithelial-mesenchymal transition (EMT). Further, we found notable differences in differentiation capacity, migration, immunosuppressive properties and secretion of immunomodulatory factors between the P0 and P5 Epilife expanded hAEC. These changes would need to be taken into account as they would have a marked impact on the potential therapeutic applications of the expanded hAEC.

Bilic et al. [4], reported that hAEC from term fetal membranes showed limited expansion in FCS supplemented DMEM/F12 medium. Since stem cells need to be expanded in xenobiotic-free media to comply with GMP for therapeutic applications [17], we expanded hAEC in commercially available xenobiotic-free and human serum containing media with rhEGF supplementation as this growth factor has been shown to induce proliferation [2], [3], [22]. Among the media tested, hAEC grew well in Epilife yielding approximately 3.7×10^7^ viable cells at P5 with a CPD of 5.2 after an initial seeding density of 1.5×10^6^ cells. Given that 50–100 million hAEC are routinely harvested from each membrane [3], [16], approximately 1.2–2.5×10^9^ cells could potentially be generated from each amnion membrane by P5. The CPD of hAEC in Epilife was low compared with human bone marrow derived MSC that have been reported to have CPD of 10–12 by P4 without losing their differentiation capacity [23]. The CCN and CPD of hAEC in Epilife was significantly lower than controls, suggesting that supplementation of cultures grown in Epilife with serum derived factors may be beneficial. Apart from EGF, hepatocyte growth factor (HGF), TGF-β, basic fibroblast growth factor (bFGF), insulin, transferrin and triiodothyronine have also been shown to promote hAEC proliferation [24]. These factors may need to be tested alone and in combination in xenobiotic-free media formulations. Since growth factors such as bFGF can induce differentiation into neuronal lineages [25] such differentiation would also need to be monitored. Human platelet lysate has also been shown to be very effective in expanding MSC while retaining their differentiation and immunosuppressive properties [26], [27]. Platelet lysate is prepared soon after blood collection and obtaining sufficient volumes for large scale culture may limit its usage.

We observed gradual morphological changes during expansion with the P0 epithelial cells changing into stromal-like cells by P3–4 in Epilife and FCS supplemented media. hAEC with an epithelial morphology were labeled with CFSE and the stromal-like cells were shown to retain the dye label. These changes were consistent with an EMT as shown by decrease in the epithelial markers E-cadherin, CD49f (integrin α6) and CK7. In contrast, MSC associated markers CD90, CD105 and CD146 increased significantly at P5 hAEC, in agreement with a previous report where hAEC were expanded in FCS containing medium [5]. We also showed that the stromal marker vimentin increased at P5. Interestingly, human embryonic stem (hES) cells grown without feeder layers have been found to change into stromal-like cells with down-regulation of E-cadherin and up-regulation of vimentin [28]. Primary hAEC display some of the pluripotency features of hES cells and it would be worthwhile investigating if culture on feeder layers could delay or prevent the changes observed in hAEC. TGF-β has been shown to induce EMT during cancer cell metastases and in chronic fibrotic diseases [29], [30]. TGF-β signaling induces Slug and Snail transcription factors that suppress E-cadherin expression [29]. The P0 hAEC secrete TGF-β and the effect of inhibiting TGF-β signaling and/or other factors linked to EMT such as tyrosine kinase receptor signaling, small GTPases, ZEB transcription factor induced by miRNA-200 family [31], [32], should be examined to determine which factor(s) play a role in changes observed during expansion of hAEC. Further, EGF also has been reported to induce EMT in some cancer cell lines [33], [34], but enhanced expression of MSC-related antigens in hAEC occur in cultures without addition of EGF [5]. In preliminary experiments addition of EGF to P0–P2 cultures did not stimulate EMT (data not shown), suggesting that EGF does not play a role.

Mature, polarized epithelial cells that undergo EMT display migratory properties [29], [35]. Interestingly, using a scratch wound assay we found that not only the stromal-like P5 hAEC were able to migrate, but that the P0 hAEC had a higher migratory capacity. hAEC lining the amnion membrane have no known migratory properties and hence factors that could regulate the migration of hAEC have not been investigated. Interactions between chemokines and their receptors, in particular CXCR4, are believed to play important roles in the migration of MSC [21]. We localized CXCR4 to hAEC lining the amnion membranes but neither P0 nor P5 hAEC expressed CXCR4. Expression of CXCR4 has been shown to decline in MSC during culture [21] and this may account for the loss of CXCR4 in the P0 cells. Since chemotactic and adhesion factors play important roles in regulating migration of stem cells to target sites of tissue inflammation and damage, it would be important to identify these factors in assessing the therapeutic applications of hAEC of the primary and expanded hAEC.

Changes in morphology and reduced differentiation capacity due to senescence have been reported in porcine and human MSC expanded in long term culture [23], [36], [37]. We found that unlike the P0 cells, P5 hAEC failed to differentiate into important endodermal lineages such hepatocytes and pancreatic cells and would limit derivation of these lineages for potential cell replacement therapies to primary hAEC and cells from early passages. However, as P5 Epilife expanded cells differentiated into osteocyte and surfactant producing alveolar epithelial-like cells, it suggests a functional alteration rather than senescence being responsible for changes in differentiation, and it would be important to determine if the expanded cells can undergo tissue specific differentiation *in vivo* as has been demonstrated for P0 hAEC [6], [9], [11]. Indeed, the TEM studies showed that the expanded hAEC had well developed rER and Golgi complexes consistent with maturation and a well developed secretory profile and not senescence. Down regulation of ES markers TRA1-60 and TRA1-81 has been reported during expansion [5] and it is possible that expression of lineage specification and differentiation pathways also alter during hAEC expansion. Expansion may also lead to selection of sub-populations within the primary isolates as notable differences in both marker expression, secretory profile and differentiation was found between FCS supplemented and Epilife expanded hAEC.

The low immunogenicity exhibited by expanded MSC from bone marrow and gestational tissue have enabled clinical trials involving allogeneic transplantation. We showed that P0 hAEC expressed low to moderate levels of HLA class IA and lack HLA class II antigens, consistent with previous reports [4], [13], [14]. Expression of HLA and the co-stimulatory molecules CD80, CD86 and CD40 is required to activate T cells and subsequent immune rejection of the transplanted cells. We found CD40 expressed by P0 cells, while both CD80 and CD86 were negligible. There were no significant differences in the expression of these antigens in the P5 hAEC. These findings may explain the survival of P0 hAEC following xeno-transplantation into immune-competent animals over prolonged periods [8], [11] and also suggest that P0 and P5 hAEC are unlikely to be rejected following xeno-transplantation.

We also examined the immunosuppressive properties of the P0 and expanded hAEC. Consistent with previous reports, P0 hAEC suppressed T cell proliferation [13], [14], [15]. The P5 hAEC also suppressed T cell proliferation, however the P0 cells were more effective at higher splenocyte ratios. The immunosuppressive properties of MSC are well established and HLA-G, IL-6 and TGF-β [38], [39], [40] among the factors known to play a role. Djouad et al [38] proposed that IL-6 secreted by MSC inhibits dendritic cell maturation and subsequently impairs T cell proliferation and induces tolerance. TGF-β1 has also been shown to inhibit T cell proliferation [39]. We found that IL-6 and TGF-β1 were secreted by P0 and expanded hAEC and these factors may partly contribute towards the suppression of T cell proliferation. On the other hand, a high percentage of P0 cells were HLA-G positive compared with P5 hAEC with cells cultured in Epilife lacking this non-polymorphic Class IB antigen. HLA-G has been shown to inhibit proliferation by binding to killer immunoglobulin-like receptors and/or immunoglobulin-like transcript on CD4^+^ and CD8^+^ T cells [41]. HLA-G is also known to modulate the cytotoxic activity of Natural Killer cells. MSC secrete other anti-inflammatory factors such as IL-10 and HGF. Interestingly, neither the P0 nor expanded hAEC secreted IL-10 or HGF.

Recent studies show that transplantation of P0 hAEC reduces tissue inflammation and fibrosis in murine liver and lungs [6], [11], although the mechanisms remain largely unknown. P5-DF expanded hAEC secreted significant amounts of MCP-1 that could induce the recruitment of monocytes and promote fibrogenesis. In addition to the immuno-modulatory effects, TGF-β1 and IL-6 play an important role in promoting fibrogenesis. Therefore, the effects of expanded P5 hAEC on tissue inflammation, monocyte chemotaxis and fibrosis would need to be tested in animal models.

In conclusion, we have shown that expanded hAEC have different properties to the primary cells. P0 hAEC may be useful for generating hepatocyte and pancreatic–like cells for therapeutic applications and expanded cells for mending bone fractures and contributing towards the alveolar epithelial cell population damaged in lung diseases. Further, the P0 cells may be more useful in suppressing tissue inflammation and fibrosis and as a treatment for autoimmune diseases and graft vs host disease where it would be important to limit T cell activation. Characterization of the transitional hAEC at passages 2–3 and testing expanded hAEC *in vivo* models would be beneficial in assessing the suitability of the expanded hAEC for cellular therapeutic applications.
